# Author Correction: A simplified mesoscale 3D model for characterizing fibrinolysis under flow conditions

**DOI:** 10.1038/s41598-023-47162-0

**Published:** 2023-11-21

**Authors:** Remy Petkantchin, Alexandre Rousseau, Omer Eker, Karim Zouaoui Boudjeltia, Franck Raynaud, Bastien Chopard, the INSIST investigators, Charles Majoie, Ed van Bavel, Henk Marquering, Nerea Arrarte‑Terreros, Praneeta Konduri, Sissy Georgakopoulou, Yvo Roos, Alfons Hoekstra, Raymond Padmos, Victor Azizi, Claire Miller, Max van der Kolk, Aad van der Lugt, Diederik W. J. Dippel, Hester L. Lingsma, Nikki Boodt, Noor Samuels, Stephen Payne, Tamas Jozsa, Wahbi K. El‑Bouri, Michael Gilvarry, Ray McCarthy, Sharon Duffy, Anushree Dwivedi, Behrooz Fereidoonnezhad, Kevin Moerman, Patrick McGarry, Senna Staessens, Simon F. de Meyer, Sarah Vandelanotte, Francesco Migliavacca, Gabriele Dubini, Giulia Luraghi, Jose Felix Rodriguez Matas, Sara Bridio, Bastien Chopard, Franck Raynaud, Rémy Petkantchin, Vanessa Blanc‑Guillemaud, Mikhail Panteleev, Alexey Shibeko, Karim Zouaoui Boudjeltia

**Affiliations:** 1 Scientific and Parallel Computing Group, Computer Science Department, University of Geneva, Geneva, Switzerland; 2 Complex System Modeling Group, Computer Science Department, University of Geneva, Geneva, Switzerland; 3 Laboratory of Experimental Medicine (ULB222), Faculty of Medicine, Université libre de Bruxelles, CHU de Charleroi, Charleroi, Belgium; 4 Department of Neuroradiology, Hôpital Pierre Wertheimer, Hospices Civils de Lyon, Lyon, France; 5 CREATIS Laboratory, UMR 5220, U1206, Université Lyon, INSA-Lyon, Université Claude Bernard Lyon 1, UJM-Saint Etienne, CNRS, Inserm, Lyon, France; 6 Department of Radiology and Nuclear Medicine, Amsterdam UMC, Location AMC, Amsterdam, The Netherlands; 7 Biomedical Engineering and Physics, Amsterdam UMC, Location AMC, Amsterdam, The Netherlands; 8 Department of Neurology, Amsterdam UMC, Location AMC, Amsterdam, The Netherlands; 9 Computational Science Lab, Faculty of Science, Institute for Informatics, University of Amsterdam, Amsterdam, The Netherlands; 10 Department of Radiology and Nuclear Medicine, Erasmus MC University Medical Center, PO Box 2040, 3000 CA Rotterdam, The Netherlands; 11 Department of Neurology, Erasmus MC University Medical Center, PO Box 2040, 3000 CA Rotterdam, The Netherlands; 12 Department of Public Health, Erasmus MC University Medical Center, PO Box 2040, 3000 CA Rotterdam, The Netherlands; 13 Institute of Biomedical Engineering, Department of Engineering Science, University of Oxford, Parks Road, Oxford, OX1 3PJ UK; 14 Cerenovus, Galway Neuro Technology Center, Galway, Ireland; 15 College of Engineering and Informatics, National University of Ireland Galway, National Center for Biomedical Engineering Science, National University of Ireland Galway, Galway, Ireland; 16 Laboratory for Thrombosis Research, KU Leuven Campus Kulak Kortrijk, Kortrijk, Belgium; 17 Laboratory of Biological Structure Mechanics, Department of Chemistry, Materials and Chemical Engineering “Giulio Natta”, Politecnico di Milano, Piazza Leonardo da Vinci 32, 20133 Milan, Italy; 18 Institut de Recherches Internationales Servier, Courbevoie Cedex, France; 19 Center for Theoretical Problems of Physicochemical Pharmacology, RAS, Moscow, Russia; 20 Dmitry Rogachev National Research Center of Pediatric Hematology, Oncology and Immunology, Moscow, Russia; 21 Faculty of Physics, Lomonosov Moscow State University, Moscow, Russia

Correction to: *Scientific Reports* https://doi.org/10.1038/s41598-023-40973-1, published online 22 August 2023

The original version of this Article contained an error in the name of INSIST investigator Simon F. de Meyer, which was incorrectly given as Simon de Meyer.

The original Article has been corrected.

